# Co-solvent polarity controlled self-assembly of tetraphenylethylene-buried amphiphile for size-regulated tumor accumulation

**DOI:** 10.1093/rb/rby010

**Published:** 2018-05-19

**Authors:** Jingsheng Huang, Yun Chen, Pengxiang Zhao, Yunlong Yu, Shiyong Zhang, Zhongwei Gu

**Affiliations:** 1 National Engineering Research Center for Biomaterials, Sichuan University, 29 Wangjiang Road, Chengdu, China; 2 Science and Technology on Surface Physics and Chemistry Laboratory, Mianyang, China; 3 College of Materials Science and Engineering, Nanjing Tech University, Nanjing, Jiangsu, China

**Keywords:** polarity control, aggregation-induced emission, size regulation, tumor accumulation

## Abstract

We report that the co-solvent polarity can precisely control the TPE-buried amphiphile 1 to self-assemble into nanoparticles (NPs) in water with size range from ∼21–32 nm to 55–68 nm to 95–106 nm. Excepted for size, these TPE-buried amphiphile fabricated NPs hold identical physical properties such as spherical shape, surface charge, and luminescent properties, and moreover, after covalent capture of the acrylate hydrophilic heads, the resulting cross-linked NPs (cNPs I–III) own excellent in vivo stability, which thus would be an ideal platform for investigating the size effects on tumor accumulation and penetration.

## Introduction

For nanomedicine, the particle size plays a vital role in accumulation and penetration of nanoparticles (NPs) entering tumors [1, 2]. Since the polymeric micelle was developed as drug delivery system (PM-DDSs) in 1980s, numerous good nanocarriers such as polymer micelles, Au nanoparticles and porous SiO_2_ nanoparticles have been used widely in drug delivery [3–6]. Although the size effects of them have been extensively studied, the best sized NPs to improve tumor diagnostics and therapeutics were still in controversial. These controversies were mainly ascribed to the different chemical structures and physical properties of NPs used for the size studies such as particle components, stability, surface charge, shape, and so on [7–13]. Such as polymer micelles, the size was regularly controlled by changing the polymer composition. For Au or SiO_2_ nanoparticles, they were always limited by biocompatibility and low drug loading contents (DLC). Therefore, for systematic investigation of the size effects in cancer drug delivery, the development of ideal platform that has exactly the same chemical structures and similar physical properties except for size would be highly valuable.

**Scheme 1:**
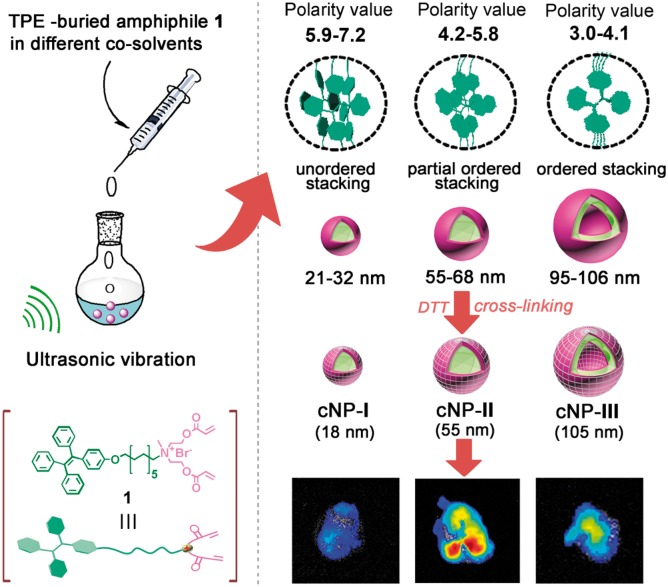
Schematic presentation of co-solvent polarity controlled self-assembly of TPE-buried amphiphile **1** for size-regulated tumor accumulation.

Fluorescent nanoparticles with aggregation-induced emission (AIE) has attracted more attention for optoelectronics, cell imaging, diagnostic sensors, and targeted drug delivery [14–17]. It has been well studied that the tetraphenylethylene (TPE) skeleton owns propeller-like conformation with one double-bond stator and four phenyl rotors [18]. In non-polar solvents, the rotors can rotate easily against the stator via the single-bond axes, while in polar solvents, the intramolecular rotation is restricted due to the physical constraint, which blocks the non-radiative transition and activates the radiative decay. The restriction of intramolecular rotation (RIR) caused luminescence enhancement is the famous effect of aggregation-induced emission [19, 20]. In this article, we report for the first time that the co-solvent polarity can precisely control the rotation degree of TPE skeleton buried in amphiphile **1**, and lead it to self-assemble into nanoparticles in water with different sizes ranging from ∼21–32 nm to 55–68 nm to 95–106 nm to investigate the size effect (Scheme 1). Importantly, excepted for size, these TPE-buried amphiphile fabricated NPs have identical physical properties such as spherical shape, surface charge, and luminescent properties, and moreover, after covalent capture of the functional hydrophilic heads, the resulting cross-linked NPs (cNPs **I–III**) hold excellent *in vivo* stability, and different linker groups are produced specific response to release drug in the tumor microenvironment [21, 22], which thus would be an ideal platform for drug delivery and investigating the size effects.

## Materials and methods

### General methods

Routine ^1 ^H NMR spectra were obtained on a Bruker AV II-400, and the chemical shifts were measured relative to D_2_O or DMSO-d_6_ as the internal reference (D_2_O: δ 4.79 ppm; DMSO-d_6_: δ 2.5 ppm). The fluorescence emission was measured by using a Hitachi F-7000 fluorescence spectrometer. The particle size and Zeta potential were measured with the Dynamic Light Scattering (DLS) Analyzer (Malvern ZetasizerNano ZS90). TEM studies were carried out using a TecnaiG2F20S-TWIN instrument, operating at 120 kV. The TEM specimens were prepared by gently placing a carbon-coated copper grid on the surface of the sample. The TEM grid was then removed, stained with an aqueous solution of 2% phosphotungstic acid, dried for 0.5 h by infrared lamps, and then subjected to TEM observation. Human liver cancer (HepG2) was obtained from Chinese Academy of Science Cell Bank for Type Culture Collection (Shanghai, China) and used for all of cell experiments and animal experiments. The cell line was grown in Dulbecco’s modification of Eagle’s medium Dulbecco supplemented with 10% (v/v) fetal bovine serum (FBS) and 1% (v/v) penicillin/streptomycin in an incubator under 5% CO_2_ at 37°C. Cell toxicity was evaluated by measuring the percentage of cell viability via the Cell Counting Kit-8 assay (CCK-8). The absorbance was then measured using a microplate reader Varioscan Flash (ThermoFisher SCIENTIFIC). The cell viability (%) was obtained according to the manufacturer’s instructions. The cellular uptake of HepG2 cells incubated with cNP-**I**, cNP-**II** and cNP-**III** was observed under confocal laser scanning microscopy (CLSM, LSM780) provided by College of Chemistry Experiment Platform Center. In vivo fluorescence images were acquired by Live Cell Imaging System (LCIS, Maestro CRi, Inc., USA). *Chemicals*: Unless otherwise noted, all reagents were obtained from commercial suppliers and used without further purification.

Deionized water was used in all aqueous experiments. Cell counter kit-8 (CCK-8) was purchased from Dojindo Laboratories (Kumamoto, Japan). BALB/c mice (18 ± 2 g, 5 weeks old) and nude mice (20 ± 2 g, 4–5 weeks old) were purchased from Dashuo Experimental Animal Company (Sichuan, China). Blood collection tubes were purchased from Chengdu Haoyi Biotechnology Company (Sichuan, China). The synthesis of compound **1** could be found in our previous report [23].

### Preparation of co-solvent polarity controlled self-assembly of tetraphenylethylene-buried amphiphile 1

Compound **1** (50 μl, 0.12 M in DMSO/Ethanol/*n*-propanol) was added dropwise into 5.0 ml of deionized water with the speed of 1.2 ml/min under ultrasonic vibration at room temperature. The resulting solution was then left to stand for 1 h and the NPs **I**–**III** formed spontaneously as a pale blue emulsion with strong Tyndall effect.

### Calculations of polarity value (*P*) of binary solvent system [24]

The polarity value (*P*_AB_) of the binary solvent system of A and B was calculated by the following formula:

$$P_{\text{AB}}=\varphi_{A}*P_{A}+\varphi_{B}*\;P_{B}$$

Where φ_A_ and φ_B_ represent solvent A and B’s volume fraction in the binary system, while PA and PB represent the pure solvent A and B’s polarity index, respectively. According to above formula, the polarity value of the binary system of 1:1 DMSO: *n*-propanol would be 5.6.

### Carboxyfluorescein leakage assay [25]

Compound **1** (50 μl, 0.12 M in co-solvent) was added dropwise into 5.0 ml of aqueous solution of 5(6)-Carboxyfluorescein (CF, 1.5 mM) under with the speed of 1.2 ml/min ultrasonic vibration at room temperature overnight. A portion (1.0 ml) of the resulting nanoparticle solution was passed through a column of Shepadex G-50 using millipore water as the eluent to remove the extravesicular CF. The nanoparticle fractions were combined and diluted to 5.0 ml with the same deionized water. The concentration of compound **1** in the solution was 0.24 mM. At this moment, the fluorescence emission at 520 nm (λex = 490 nm) was recorded. After that, 20 µl of Triton X-100 (1%) was added to lyse the vesicles, and the fluorescence emission (λex = 490 nm) was measured again. The leakage assay results are shown in Supplementary Fig. S2.

### Typical preparation of cross-linked nanoparticles I–III (cNPs I–III)

To 5.0 ml aqueous solution of NPs (1.2 mM), dithiothreitol (DTT, 1.1 mg, 7.2 mmol) was added under nitrogen atmosphere. The reaction mixture was stirred at 40°C for 16 h and dialyzed against deionized water for 2 days (Spectra/Pore, MWCO 1000) to get the corresponding cNPs as a pale blue solution.

### Stability test of cNPs I–III and NPs I–III *in vitro*

Briefly, the cNPs ([1] = 1.0 mg/ml) was diluted to the concentrations of 1000, 100, 20, and 10 μg/ml, respectively. Afterwards, the particle sizes of above solutions were recorded by DLS to evaluate its stability.

The FBS stability of NPs **I–III** and cNPs **I–III** was investigated by incubation with 10% (V/V) FBS. Briefly, 9 ml of NPs and cNPs ([1] = 1.2 mM, in deionized water) were mixed with 1 ml FBS, respectively. The particle sizes at 0 h and over 24 h of incubation at 37°C were recorded to evaluate their stability.

### Cellular uptake evaluation by confocal laser scanning microscopy

HepG2 cells (5 × 104 cells/ml) were seeded in a Φ = 35 mm glass Petri dish and incubatedat 37°C/5% CO_2_ for 24 h. Subsequently, the cells were cultured with equal concentration of cNPs **I–III** ([1] = 2.5 μg/ml, λex = 405 nm, λem = 480 nm), at 37°C for 1, 3, and 6 h, respectively. The culture medium was removed and the cells were washed three times and another 1 ml PBS was added. The cellular uptake was then observed under confocal laser-scanning microscopy. The CLSM test of cNP-II, DOX@cNP-**II** and free DOX•HCl ([DOX] = 2.5 μM, [1] = 10 μM) were similar to the above procedures.

### Cytotoxicity assay

In vitro cytotoxicity was assessed by the Cell Counting Kit-8 assay (CCK-8). Briefly, HepG2 cells (5000/well) were seeded in 96-well culture plates and incubated at 37°C/5% CO_2_. After 24 h, culture media was removed and fresh media (200 μl) containing cNPs **I–III** which ranging from 10 to 200 μg/ml was added to each well, separately. Cells without any treatment were set as control. After 24 h, culture media was removed and fresh media (100 μl) containing CCK-8 (10 μl) was added to each well and the plates were incubated at 37°C for another 2 h. Then, the absorbance at 450 nm of each sample was measured using amicroplate reader Varioscan Flash. The Cell viability of DOX@cNP-**II** and free DOX•HCl with different concentrations from 0.05 to 20 μg/ml were similar to the above procedures.

### *In vivo pharmacokinetic study* [26]

BALB/c mice (∼20 g, 5 weeks old) were randomly divided into three groups (*n* = 3) and treated with cNPs **I–III** ([1] = 5.0 mg per kg-1) via tail vein. The blood samples were taken from the eye socket at the 3 min, 0.5 h, 1 h, 2 h, 6 h, 12 h and 24 h time points after injection. The blood (about 0.6 ml) samples were centrifuged (3000 g, 10 min) at 4°C. A 100 μl aliquot of plasma was treated with 50 μl of HCl (5 M) to break the sensitive bond and incubated for 3 h at 50°C. Then 50 μl of NaOH (1.0 M) was added and incubated for 15 min at room temperature. The mixed 200 μl solvents (acetonitrile/methanol = 1: 1, v/v) was extracted twice and separated by centrifugation (10 000 g, 5 min). The organic phase was collected and pooled. Then, the organic phase was dried by Termovap Sample Concentrator. At last, the samples was dissolved in 1 ml water and directly examined by using fluorescence spectroscopy. A standard curve was made by adding various concentrations of cNPs to the plasma. All samples can be calculated according to the standard curve. Pharmacokinetic parameters were analyzed using the pharmacokinetic software DAS 3.0 (Mathematical Pharmacology Professional Committee, People’s Republic of China) by fitting to the two-compartment model.

### *In vivo* tumor accumulation study [27]

The dilution stability of cNPs **I–III** was evaluated by diluting the concentrations of **1** below its CACs in corresponding co-solvent. The tumor accumulation was investigated by the HepG2 xenograft model. 1 × 10^6^ HepG2 cells suspended in 100 μl of PBS were inoculated subcutaneously in 4-week-old female nude mice. After 2−3 weeks, the solid tumors reached about 200 mm^3^. Then, the mice were randomly separated into three groups, and injected intravenously via tail vein with cNP-**I**, cNP-**II** and cNP-**III**, respectively (5 mg kg^−1^). At 12 h postinjection, major organs (heart, liver, spleen, lung, and kidney) and tumor were excised and washed with saline (3 × 10.0 ml) for semi-quantitative analyses by detecting their fluorescence signals. Fluorescence signals were collected by a fluorescence imaging system.

### Typical preparation of cross-linked nanoparticles loading hydrophobic DOX (DOX@cNP-II)

The NP-**II** was prepared by thiol-acrylate Michael addition. Briefly, the solution of small-molecule micelles (4.0 ml, [1] = 1.2 mM) was added dithiothreitol (DTT, 2.6 mg, 0.02 mmol), respectively and prepared hydrophobic DOX (1 mg, in 200 μl ethanol). Then, the mixture was stirred overnight and dialyzed against deionized water for 1 days (Spectra/Pore, MWCO1000) to get the DOX@cNP-**II** as a red solution. The some precipitate could be removed by centrifugation (2800g) in 4 min. For calculation of drug loading content (DLC), the DOX@cNP-**II** were destroyed in an acid environment (0.1 M HCl), followed by freezedrying, and redissolved in DMSO. The fluorescence intensity of the DOX was detected (Ex = 480 nm) in DMSO solution using a preestablished calibration curve with various DOX concentrations. The DLC was calculated as follows:

DLC (wt %)=(weight of loaded DOX/total weight of DOX@cNP−II)×100%

### *In vitro* release assay

Each 1.0 ml of the aliquot sample, DOX@cNP-**II** was added into a dialysis bag (MWCO 6000−8000) and dialyzed against 25.0 ml of different buffers (PBS 7.4 and ABS 5.0) with gentle shaking (100 rpm) at 37°C. At predetermined periods, 1.0 ml of the solution was collected from the corresponding different reservoirs and the samples were detected by fluorescence spectra. To keep a constant volume after each sampling, 1.0 ml of corresponding buffer solution was added to the reservoir. The data of the release experiment were averaged over three times.

## Results and discussion

### Co-solvent polarity controlled self-assembly of TPE-buried amphiphile 1

**Figure 1:**
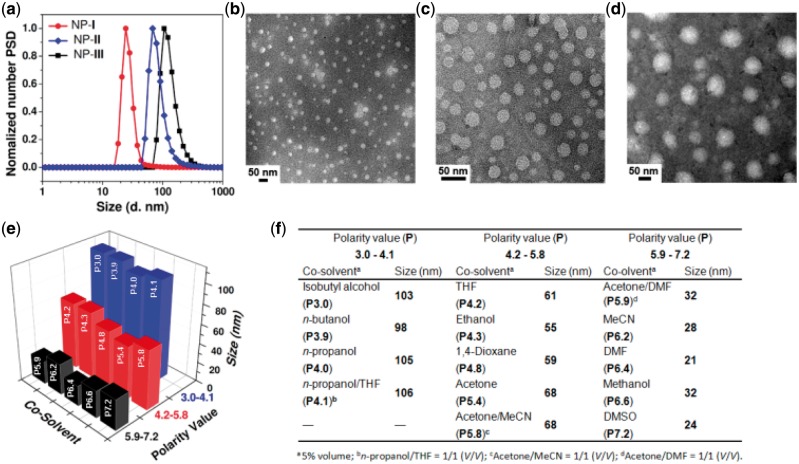
Characterization of the co-solvent polarity controlled self-assembly of TPE-buried amphiphile **1** in water. (**a**) Distribution of the hydrodynamic diameters of compound **1** fabricated NPs **I–III** in DMSO, ethanol, and *n*-propanol, respectively. (**b**), (**c**) and (**d**) TEM images of compound **1** fabricated NPs **I–III** in DMSO, ethanol and n-propanol in sequence. (**e**) Map of hydrodynamic diameters of TPE-buried amphiphile **1** fabricated NPs in various co-solvent polarities. (**f**) Quantitative values of hydrodynamic diameters of compound **1** fabricated NPs in various co-solvent polarities

The solvent polarity controlled self-assembly is easy: As compound **1** in co-solvent was added dropwise into deionized water under ultrasonic vibration at room temperature (co-solvent: H_2_O = 1: 20, V/V), a light blue solution with strong Tyndall effect was obtained, a characteristic of the NPs formed. Three typical co-solvents, dimethyl sulphoxide (DMSO), ethanol, and n-propanol, were chosen to study this polarity controlled self-assembly behavior, where their polarity value were 7.2, 4.3 and 4.0, respectively. As shown in Fig. 1a, when the high polar DMSO was adopted, the dynamic light scattering (DLS) measurement showed the formation of NPs with the diameter of ∼24 nm. As the co-solvent was changed to a less polar ethanol, the nanoparticle size increased to ∼55 nm. Moreover, when a more non-polar co-solvent *n*-propanol was employed, the size of the formed NPs further increased to ∼105 nm. Transmission electron microscopy (TEM) presented that all three NPs had a spherical-like morphology (Fig. 1b–d). Notably, the particle size determined from TEM is a little bit smaller than that from DLS. This result is reasonable to assume that the DLS assay determines the hydrodynamic diameter with fully hydrated particles in solution while the TEM assay measures the dry samples in the collapsed state. Further carboxyfluorescein (CF) leakage assay indicated that the NPs with the diameters of ∼24 and 55 nm would be a micellar structure, while the NPs with the size of ∼105 nm prefer to be a vesicular structure (See Supplementary Fig. S2 and Experimental Section for details) [23, 25].

After discovery of the importance of co-solvent in self-assembly, the assembly behavior of compound **1** was systematically investigated in different solvent systems, and the results are listed in Fig. 1e and f. We were surprised to find out that the assembly of the amphiphile **1** was precisely controlled by co-solvent polarity: When the co-solvent polarity ranged from 3.0 to 4.1, the spherical micelles with diameter of ∼21–32 nm were obtained. When the polarity located between 4.2 and 5.8, the nanoparticles with diameter of ∼55–68 nm were formed. As the solvent polarity was adjusted to ∼5.9–7.2, the large vesicles with the size of ∼95–106 nm were achieved. We rationalized that the polarity here would play a role in regulating the rotation degree of the propeller-like TPE skeleton, which further influenced the intermolecular π–π stacking of the amphiphile **1** (Scheme 1) [28] and resulted in the formaiton of different sized NPs. It is worth emphasizing that the co-solvent polarity controlled self-assembly is independent to the solvent type. As long as the polarity value is known, even if the use of a random binary solvent system, its assembly behavior can still be predicted in advance. For instance, compound **1** forms 24 nm micelles in DMSO and 105 nm vesicles in *n*-propanol, while a binary system of 1: 1 DMSO: *n*-propanol (V/V) was employed as the co-solvent, whose polarity is 5.6 calculated from the polarity index formula (See Experimental Section for details) [24], a new NPs with the size of 57 nm were obtained (Supplementary Fig. S3), a size located in the polarity value of 4.2–5.8 determined size range (55–68 nm). Notably, in the absence of co-solvent, compound **1** cannot form reproducible self-assemblies determined by DLS due to its low dispersibility in water.

### Physicochemical properties of the TPE-buried amphiphile fabricated NPs (NPs **I-III**) and cross-linked NPs (cNPs **I-III**)

**Figure 2:**
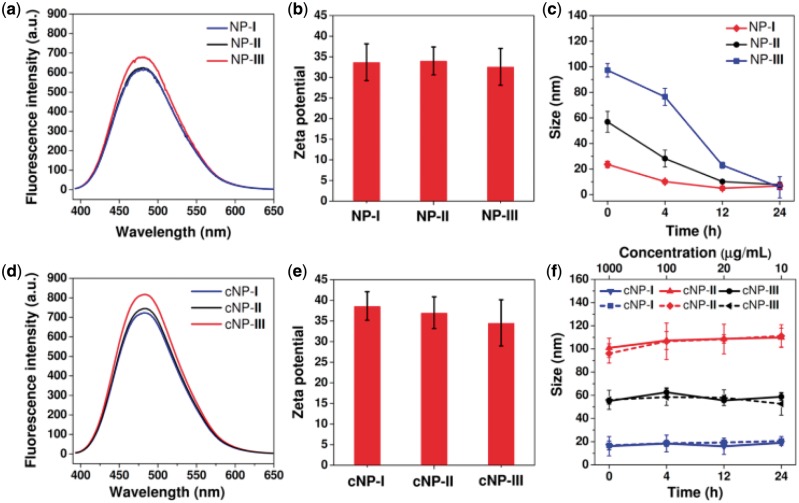
Physicochemical properties of compound **1** fabricated NPs (NPs **I–III**) and cross-linked NPs (cNPs **I–III**). (**a**) The emission spectra of NPs **I–III** excited at 373 nm. [1] = 1.2 mM. (**b**) Zeta potentials of NPs **I–III**. (**c**) Particle sizes of NPs **I–III** after incubation with 10% FBS at 37°C over time ([1] = 200 μg/ml). (**d**) The emission spectra of cNPs **I–III** excited at 373 nm. (**e**) Zeta potentials of cNPs **I–III**. (f) Particle sizes of cNPs **I–III** after incubation with 10% FBS at 37°C over time (solid line, [1] = 200 μg/ml) and after dilution (dash line), respectively

Excepted for size, these TPE-buried amphiphile fabricated NPs hold very close physical properties such as luminescence and zeta potentials (Fig. 2a and b). The luminescence intensity had a little difference result from its stress of TPE stack enhances [29]. As shown in Scheme 1, the cNP **I–II** shows different π–π stack and intramolecular rotation to enhance stress of TPE stack and the liposome-like cNP **III** with strongest stress of TPE stack in narrow shell. Unfortunately, since these NPs are assembled by small amphiphile, they decomposed easily in complex serum environment (Fig. 2c). To address this issue, these NPs were covalently captured by Michael addition in the presence of the bridging-unit dithiothreitol (DTT) (See Experimental Section for details) [30–32]. The successful capture of the NPs was proven by both the disappearance of the double bonds and broadened proton signals in ^1^H NMR spectra (Supplementary Fig. S4). The DLS and TEM measurements show that the cross-linked NPs (cNPs **I–III**) kept similar size and morphology to these before cross-linking (Supplementary Fig. S5). Notably, in addition to the maintenance of very close luminescent properties and zeta potential (Fig. 2d and e), the stability of the cNPs were greatly improved as expected. As shown in Fig. 2f, when the cNPs was diluted in water so that the concentration of (cross-linked) **1** were below their CAC (Supplementary Fig. S1), the particle size remained almost unchanged. Moreover, even after incubation in 10% FBS for 24 h the size of the cross-linked NPs remained stable, suggesting the tolerance of cNPs to the complex bloodstream. These superiorities make the cNPs **I–III** fit very well to be the platform for studying the size effect on tumor aggregation and penetration.

### *In vitro* cellular uptake of the three sized cross-linked NPs (cNPs **I-III**)

We first investigated the effect of three sizes of cross-linked NPs, for example, 18, 55, and 105 nm, on *in vitro* cellular uptake. Briefly, the HepG2 cells were cultured with equal concentrations of the cNPs **I–III** (60 μM) at 37°C for 1, 3, and 6 h, and the confocal laser scanning microscopy (CLSM) was used to compare the cellular uptake efficiency. The experimental results show that the cell uptake of the cNPs is nearly size independent. As exhibited in Supplementary Fig. S6, there is no obvious uptake difference between 18 and 55 nm cNPs at every time point. Even for the 105 nm cNPs, the cell uptake rate can just a little bit lower than that of the other two. This *in vitro* result can actually be easily understood considering that these cNPs are all positive charged. We further conducted cell viability assays to check the cytotoxicity of the cNPs **I–III**. It was found that these materials exhibited negligible cytotoxicity up to a concentration of 200 μg ml^−1^ in HepG2 cells (Supplementary Fig. S7).

### *In vivo* pharmacokinetic and tumor accumulation study

**Figure 3:**
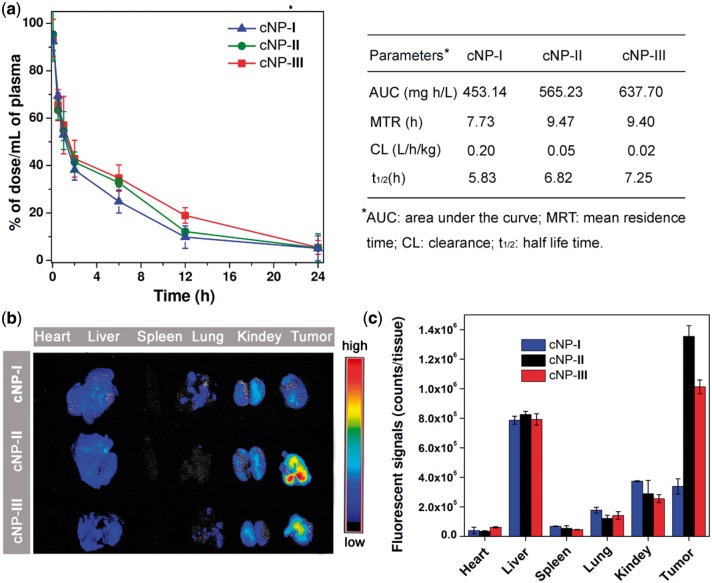
*In vivo* investigation of three sized cross-linked NPs (cNPs **I–III**). (**a**) Pharmacokinetic profiles after intravenous injection of cNPs **I–III** in BALB/c mice (5 mg compound **1** per kg). (**b**) Ex vivo fluorescence images of tissues of the HepG2 tumor bearing nude mice after 12 h intravenous injection of cNPs **I–III**. (**c**) Quantitative analysis of the fluorescence intensity of tissues at 12 h post injection (mean ± SD, *n* = 3)

The *in vivo* pharmacokinetic investigation disclosed that the blood clearance of the cNPs is size dependent. As illustrated in Fig. 3a, when the cNPs **I–III** were intravenously injected into the BALB/c mice at the dose of 5.0 mg per kg^−1^, the blood clearance of the cNPs became slower as the size increased from 18 to 105 nm. The 18 nm micelles were cleared quickly from the bloodstream, but the blood clearance of 105 nm particles was slow. Quantitatively, compared with the 18 nm group parameters, area under the curve (AUC) of 453.14 and half-life (t_1/2_) of 5.83 h, the 55 nm NPs increased the AUC to 565.23 (1.25-fold) and enlarged the t_1/2_ 6.82 h (1.17-fold). Moreover, the AUC and t_1/2_ of 105 nm cNPs were 637.70 and 7.23 h, up to 1.41- and 1.24-fold increase to those of the 18 nm cNPs, respectively.

To evaluate the accumulation behavior of the three sized cNPs **I–III** in the tumor tissue *in vivo*, the HepG2 tumor bearing nude mice were developed and intravenously administered by 18, 55 and 105 nm cNPs at an equivalent dose of 5 mg of 1 per kg. The tumor and major organs of all groups were then excised at 12 h post injection. Figure 3b shows the ex vivo fluorescence images of each organs. Interestingly, one can find that the large 105 nm cNPs with prolonged blood retention time did not lead to better tumor accumulation and penetration. Instead, the medium sized 55 nm particles exhibited higher tumor tissue aggregation at the selected time point. The further quantitative results showed that the fluorescence signals of ∼1.4 × 10^6^ counts of 55 nm cNPs are accumulated in the tumor site at the 12 h post injection, which is 4.1 times than that of 18 nm cNPs and 1.4 times than that of 105 nm group (Fig. 3c). This result clearly demonstrates that the tumors of the 55 nm group possess higher levels of cNPs compared with that of 18 nm and the 105 nm groups.

Collectively, the above outcomes indicated that the blood circulation and tumor accumulation are the two intrinsically conflicting attributes of using nanoparticles for cancer treatment: The larger NPs are optimal for slower blood clearance, but they are too large to diffuse into tumor tissues composed of tightly packed cells in a dense extracellular matrix. Smaller NPs show much better tumor penetration, but typically suffer from short half-life time in blood circulation. Therefore, finding the right size NPs that can take into account both the blood circulation and tumor penetration would be a key issue to determining how far a drug delivery system can move forward. Our study clearly disclosed that under the consistently physical properties, ∼55 nm is a relatively ideal size to balance the two conflicts.

### *In vitro* tests of DOX@CNP-II

**Figure 4:**
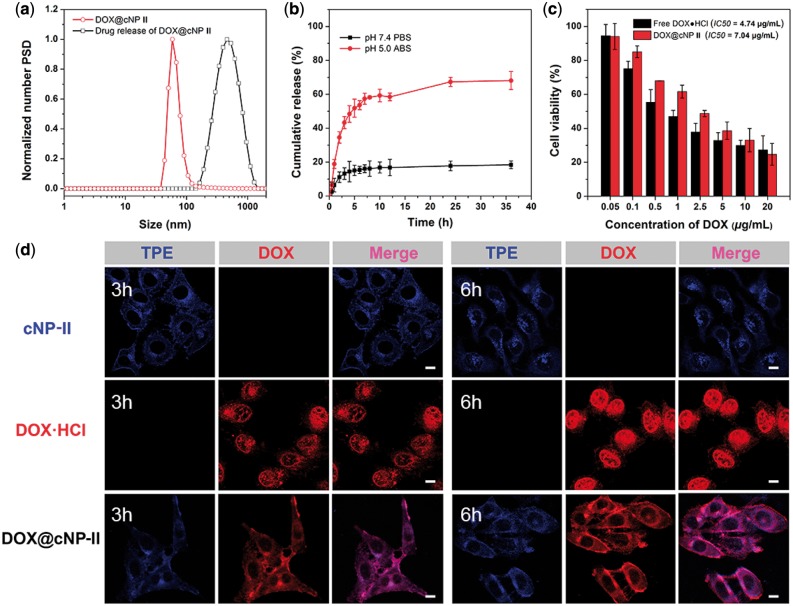
*In vivo* investigation of cross-linked NPs **II** drug delivery. (**a**) DLS of DOX@cNP-**II** before or after release DOX. (**b**) *In vitro* DOX release of pH-labile DOX@cNPs **II** under pH = 7.4 (PBS buffer) and pH = 5.0 (acetate buffer) at 37°C over time. (**c**) Cell viability of free DOX•HCl and DOX@cNP-**II** against HepG2 liver cells after incubation for 24 h at 37°C with a series of concentrations. (mean ± SD, *n* = 5). (**d**) CLSM images of HepG2 cells treated with free DOX•HCl, cNP-**II** and DOX@cNP-**II** for 3 and 6 h. For each panel, the fluorescence of TPE in cells (blue), the fluorescence of DOX in cells (red) and merge fluorescence (pink). The scale bars are 10 μm in all images ([DOX] = 2.5 μM, [1] = 10 μM)

The NP-**II** with optimal size entraped hydrophobic DOX (drug loading content, DLC = 12%) which is higher than polymer micelles because of π–π interaction between drug and TPE moieties. DOX@NP-**II** was cross-linked by DTT which can respond to specific tumor microenvironment. In Fig. 4a, the DOX@cNP-**II** shows similar size to cNP-**II** which is 55 nm. Under pH 5.0, the platform was disassembled and the drug was released. As shown in Fig. 4b, DOX@cNPs **II** was released much slower under pH 7.4 vs pH 5.0. The pH-dependent DOX@cNPs **II** released the loaded DOX up to 68% under acid environment corresponding to Fig. 4a. In vitro cytotoxicity of DOX@cNP-**II** and free DOX•HCl towards HepG2 liver cancer cells was evaluated by CCK-8 assay. Cell viability was examined after 24 h treatment when the highest concentration of DOX reached 20 μg/ml in Fig. 4c. And the IC50 of DOX@cNP **II** (7.04 μg/ml) was higher than free DOX (4.74 μg/ml) because DOX@cNP-**II** could overcome side effect of free drug. For further direct observation subcellular locations by Confocal Laser Scanning Microscope (CLSM), the cNP-II, free DOX•HCl and DOX@cNPs-**II** were incubated with HepG2 cells for 3 and 6 h. The blue fluorescence attributed to TPE moiety and red fluorescence owning to DOX structure as shown in Fig. 4d. The free DOX•HCl entered cell through diffusion whicle the cNP-II, DOX@cNP-**II** were uptook by endocytosis. According to CLSM study, free DOX had entered the cell nucleus at 3 or 6 h incubation and more amounts of DOX@cNP-**II** entered cytoplasm after 3 vs 6 h incubation. The DOX@cNP-**II** only partially entered the cytoplasm while the loaded DOX was released at 6 h incubation. The result hinted that the nanocarrier system could be directly observed the process of drug release during the whole process of drug delivery.

## Conclusions

In conclusion, a new class of nano-platforms by utilizing the co-solvent polarity controlled self-assembly of TPE-buried amphiphile has been established to study the size effects for efficient anticancer drug delivery. Excepted for size, the resulting NPs hold exactly the same chemical structure and very close physical properties. The *in vivo* investigation showed that the medium 55 nm would be the most effective size to achieve the best tumor accumulation of NPs. Notably, although only three sized NPs were prepared here, considering the structural tunability of amphiphilic molecules, various sized NPs could be obtained by using the solvent polarity controlled self-assembly, which would be highly promising in ascertaining the size effects in cancer drug delivery and imaging.

## Supplementary Material

Supplementary Information
