# Supplementary material for: Co-solvent polarity controlled self-assembly of tetraphenylethylene-buried amphiphile for size-regulated tumor accumulation
Source: Regen Biomater. 2018 May 19;5(5):275–82. doi: 10.1093/rb/rby010 (PMC6184576; doi:10.1093/rb/rby010)
Supplement: Supplementary Information [file rby010_supporting_information.pdf]

Supporting Information

Co-Solvent Polarity Controlled Self-Assembly of Tetraphenylethylene-Buried Amphiphile for Efficient Anticancer Drug Delivery

Jingsheng Huang,[†] *Yun Chen,*[†] *Pengxiang Zhao,*[‡] *Yunlong Yu,*[†] *Shiyong Zhang,*[*,†] *and Zhongwei Gu*[†,§]

[†] National Engineering Research Center for Biomaterials, Sichuan University, 29 Wangjiang Road, Chengdu 610064, China

[‡] Science and Technology on Surface Physics and Chemistry Laboratory, Mianyang 621907, China.

[§] College of Materials Science and Engineering, Nanjing Tech University, Nanjing 210009, Jiangsu, China.

[*] To whom correspondence should be addressed. S. Zhang, E-mail: szhang@scu.edu.cn; Phone: +86-28-85411109. Fax: +86-28-85411109.

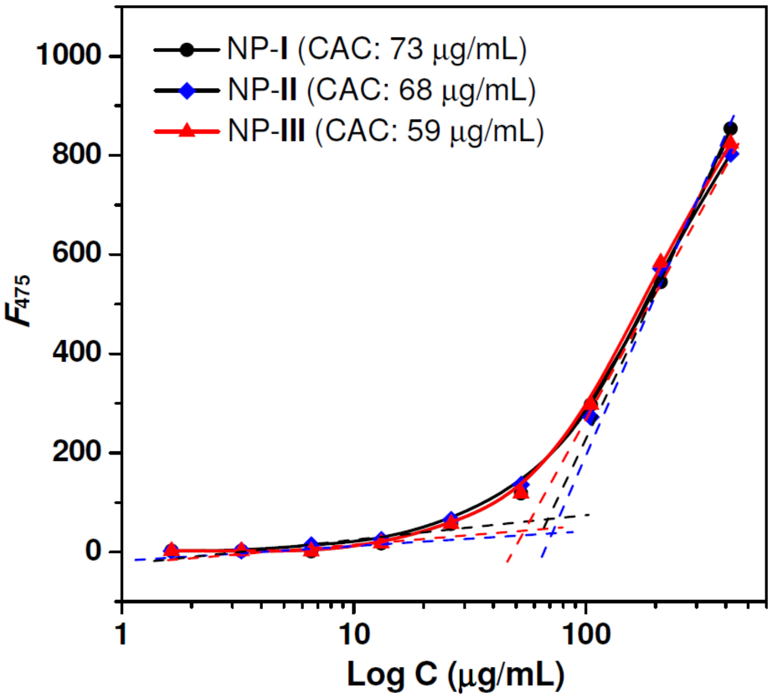

Figure 1S. Emission intensity at 475 nm (F_{475}) of NPs **I**-**III** as a function of concentrations of compound **1** in the presence of co-solvent DMSO (NP-**I**), ethanol (NP-**II**), and *n*-propanol (NP-**III**), respectively. λ_{ex} = 373 nm.

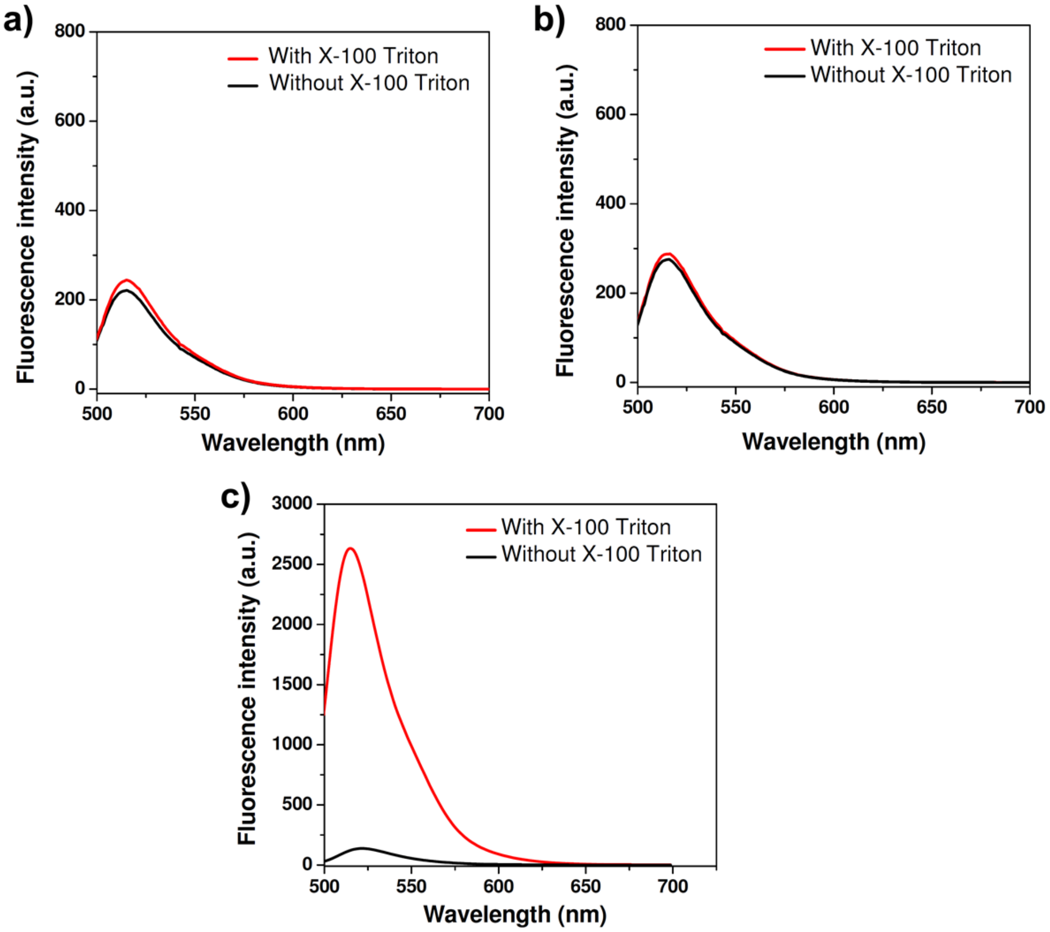

Figure 2S. Comparison of the fluorescence spectra of TPE-buried amphiphile **1** fabricated NPs in the presence of co-solvent DMSO (a), ethanol (b), and *n*-propanol (c) before and after adding Triton X-100 (1% v/v). λ_{ex} = 373 nm. The performance of fluorescence at 523 nm before and after Triton X-100 addition suggested that the NPs in the presence of co-solvent DMSO and ethanol would be a micellar structure, while the NPs formed in *n*-propanol prefer to be a vesicular structure.

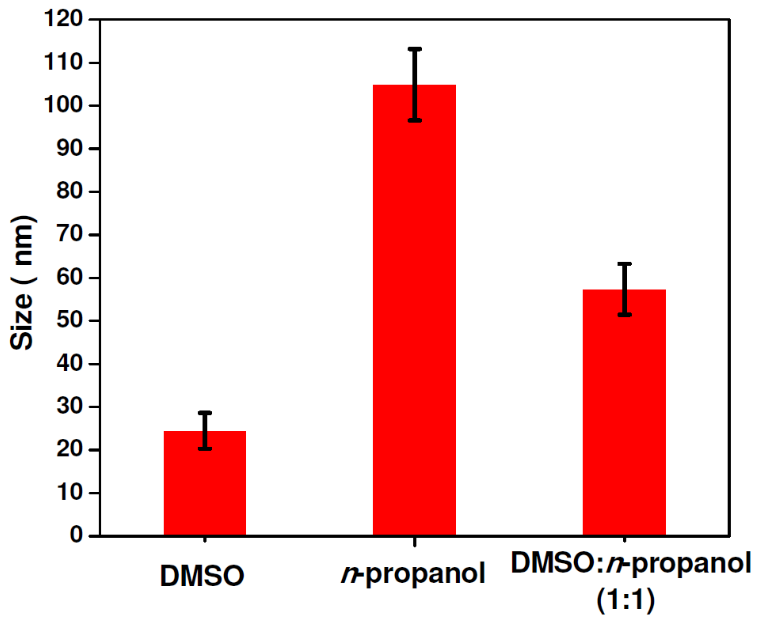

Figure 3S. Comparison of particle sizes of NPs fabricated by TPE-buried amphiphile **1** in the co-solvent of DMSO, *n*-propanol and 1:1 of DMSO : *n*-propanol (V/V), respectively.

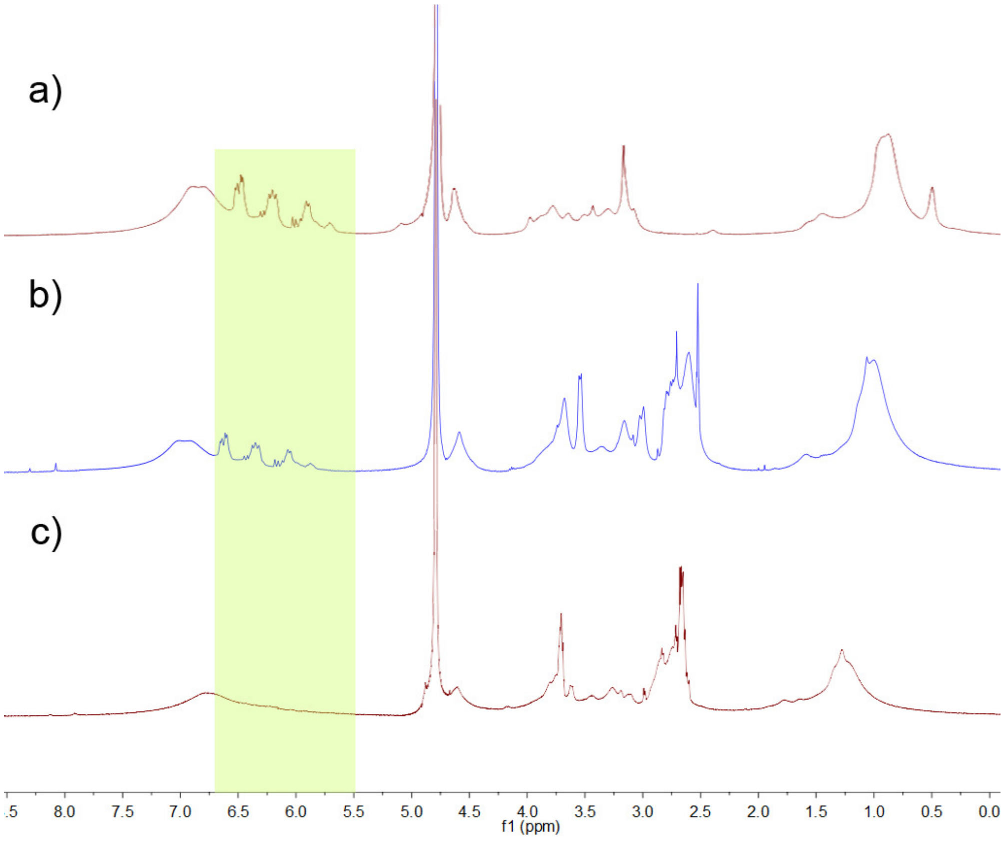

Figure 4S. ^{1}H NMR spectra of a 6.0 mM deuterated aqueous solution of TPE-buried amphiphile **1** fabricated NP-**I** in DMSO-d_6 (a), after addition of 1.0 equiv of DTT, and after cross-linking (c).

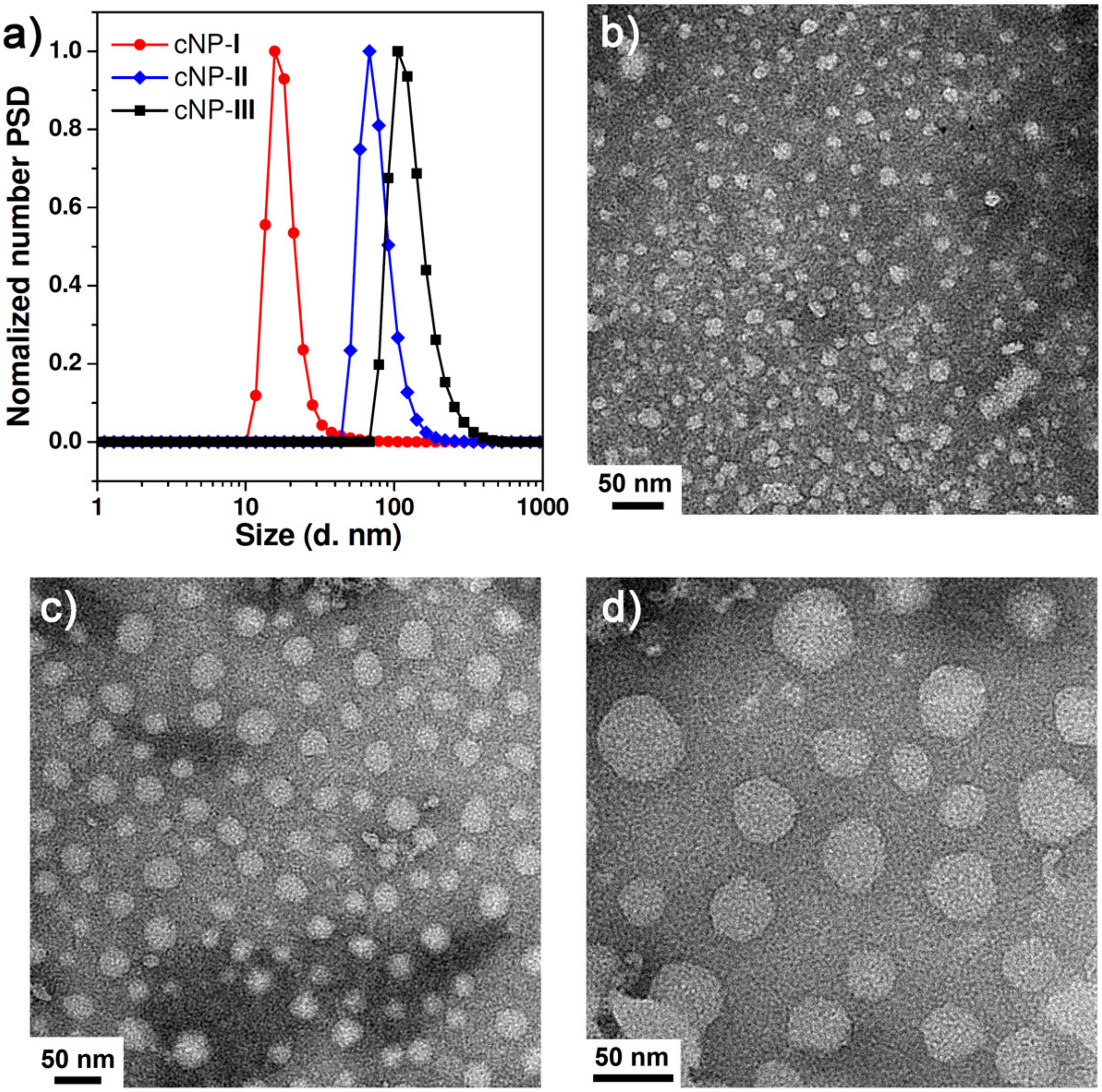

Figure 5S. (a) Distribution of the hydrodynamic diameters of cross-linked NPs **I-III** formed by TPE-buried amphiphile **1** in the presence of co-solvent DMSO, acetone, and *n*-propanol, respectively. (b), (c) and (d) TEM images of cross-linked NPs **I-III** formed by amphiphile **1** in DMSO, acetone, and *n*-propanol in sequence.

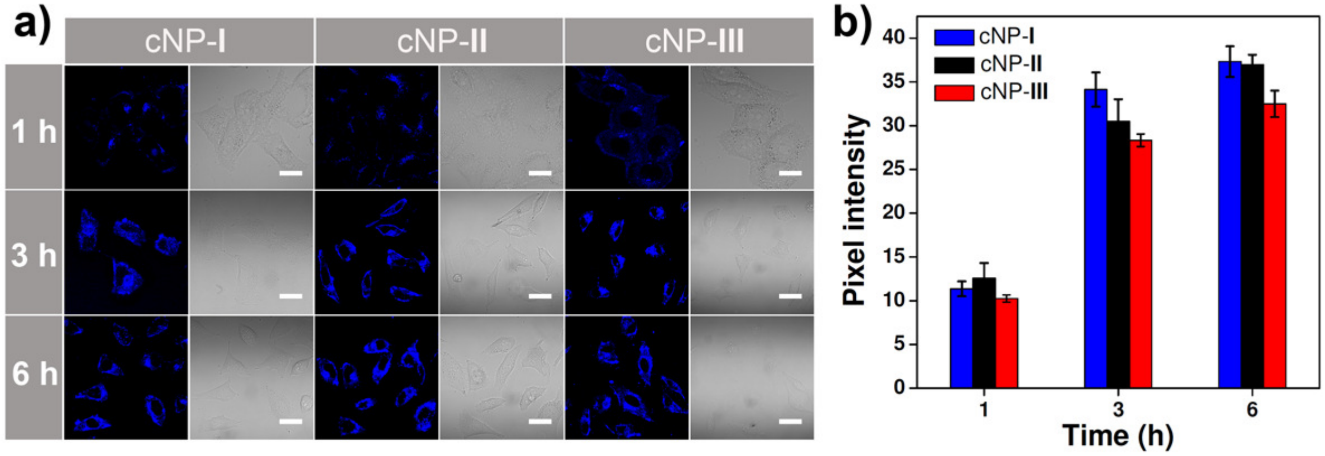

Figure 6S. *In vitro* investigation of three sized cross-linked NPs **I-III**. (a) CLSM images of HepG2 cells treated with cNPs **I-III** for 1, 3, and 6 h. Images from left to right show the fluorescence of TPE in cells (blue), the bright field and overlays of the two images, respectively. The scale bars are 25 μm in all images. (b) Quantitative analysis of the fluorescence intensity of cells at 1, 3, and 6 h post injection, respectively. [**1**] = 60 μM.

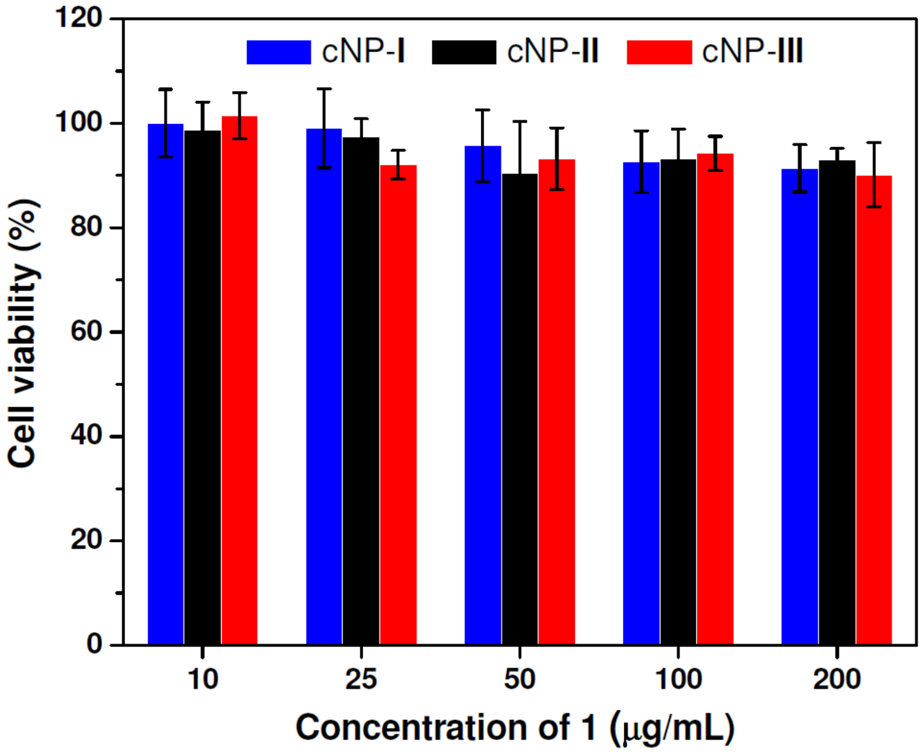

Figure 7S. Cytotoxicity of cross-linked NPs (cNPs **I-III**) against HepG2 cells for 24 h incubation.
